# Hints on the Treatment of Errors of Refraction

**Published:** 1907-06-15

**Authors:** 


### Ophthalmology.

### HINTS ON THE TREATMENT OF ERRORS OF REFRACTION.

### I. THE NECESSITY FOR A KNOWLEDGE OF THE PROCESS BY THE GENERAL PRACTITIONER.

The prescribing optician has come to stay. That fact is evident from the steady increase of opticians' shops, now rapidly becoming as numerous as those of bootmakers, hosiers, or other purely trade concerns. These spectacle shops make no pretence as to their standing; there is no suggestion that glasses will only be supplied upon the prescription of the medical man, but the appeal is frankly to the public direct. Crystals, models of the human eye and its muscular apparatus, parallel lines set in all axes to catch the eye of the astigmatic, patent forms of glass, which by their intrinsic merit are credited with curing or relieving all the eye-strain in the world, booklets on the dangers of uncorrected ocular defects, and the free estimation of errors of refraction are all shop-window baits to lead up to the sale of a pair of spectacles or pince-nez, from which the tradesman makes his profit. The public is attracted and patronises the shops, as is evident from their multiplication and success, and it is certain that the medical profession will be as powerless to suppress them as it is to suppress the sale of quack or patent medicines.

The causes of the development of this new industry are numerous. The principal one is the strain put upon the eyes of the nation by the universal education of all classes and the output of daily and weekly reading matter. The strain of modern civilisation falls almost entirely upon the visual apparatus and the mental equipment of the human body, and slight imperfections of the eye which formerly were not noticed now become a serious handicap to all those who live by the use of their eyes and brains. Newspapers, books, typewriting, shorthand, and artificial light in offices and schools all throw a strain upon the eyes which must call attention to even slight ocular imperfections. The result is that people are increasingly in need of accurate glasses, and must obtain them somewhere at a price which is within their reach. The poorer classes crowd into the out-patient departments of the special and general hospitals, where it is now a matter of common observation that mechanical defects of the eye constitute about 80 per cent. of all the cases seen. The middle classes are debarred by the almoners and inquiry officers of the hospitals from availing themselves of charitable relief, and they have no alternative but to consult their own medical practitioner, or seek the advice of the consultant ophthalmic surgeon. The great majority of medical men have no knowledge whatever of the treatment of errors of refraction; it is not a subject of the curriculum of the ordinary medical student; it requires some skill and considerable experience to obtain even moderate proficiency, and therefore the ordinary doctor avoids the whole subject from lack of knowledge. His unfortunate patients cannot afford the guinea or two guinea fee of the ophthalmic surgeon, so that they go of necessity and seek the advice of the prescribing optician, who will test the error of refraction for nothing in the hope of making his trade profit from the sale of the necessary, or unnecessary, glasses. Here it must be remembered that as far as the mechanical estimation of a purely mechanical defect is concerned the prescribing optician may be quite as successful as the medical man, and in those cases in which the ocular defect is unaccompanied by any general medical condition he may do no harm and may relieve his customer. As is the way of the general public in these cases, they are always more grateful to the unqualified practitioner, and think more highly of his services, than of those of the more expensive, but better qualified, ophthalmologist.

It remains, therefore, to consider how the competition of the prescribing optician can be met, and in what way his practice and method must be inferior to that of the practitioner; and how the public may be assured that by consulting the properly qualified medical man they really obtain better value for their money, for this is the only argument which really appeals to them. The first essential is that, if possible, all general practitioners should be able to treat with some ability and confidence the simpler and commoner errors of refraction. By doing this they will be able to ensure that their patients will obtain the necessary prescription for glasses at a moderate fee, and will be able to claim, very properly, that as they are not personally interested in the sale of glasses their advice is more honest than that of the most astute or efficient tradesman. The practitioner has also the power, denied to the optician, of ordering the instillation of such drugs as atropine or homatropine, without which it is impossible in the great majority of cases to obtain a proper estimation of any visual error. He can, in addition, diagnose corneal and lental opacities, defects in the transparency of the visual media and organic disease in the optic nerve, choroid, or retina, which may show at once that the visual defect is not one that may be properly treated by glasses. He has also the necessary qualification to correlate the patient's visual defect with his general medical condition, and is thus in a position to make a more accurate estimation of the patient's needs than the mere mechanic. By all these means, therefore, even in the simplest cases, he is enabled by his medical skill to give the patient a clean bill of health or the emphatic assurance that his eyes are sound with the exception of a slight mechanical defect in the curvature of some of the surfaces.

It is with the idea of assisting the practitioner to acquire the necessary knowledge and skill in the treatment of errors of refraction that articles will appear in The Hospital from time to time upon the subject. They will not profess in any way to supplant the ordinary text-books, and will premise an elementary knowledge of the optics required for the purpose. Their aim will be to assist the medical man by practical hints as to the difficulties of the process and the pitfalls that may be met with, and to point out to him how, by a little patience and skill, he may exercise the whole of his professional ability to the advantage of those who suffer from headaches or other results of visual defects, and, lastly, and by no means leastly, keep them out of the hands of the specious tradesman. The next article will deal with the methods of testing the visual acuity of patients, suggestions as to the routine to be adopted in all cases, and hints as to the most desirable method of treatment of children, young adults, and older people. Other articles will follow upon the method of the estimation of errors of refraction by retinoscopy, specimen cases for treatment, muscular errors, the use of prisms, and the treatment of squints.

